# An innovative technique for detecting the caudal end of occluded inferior petrosal sinus in cavernous arteriovenous fistula using intravascular ultrasonography—technical note

**DOI:** 10.1007/s00234-015-1530-8

**Published:** 2015-04-24

**Authors:** Shigeru Yamauchi, Akimasa Nishio, Yoshinobu Takahashi, Kimito Kondo, Taichiro Kawakami, Yuzo Terakawa, Yutaka Mitsuhashi, Kenji Ohata

**Affiliations:** Department of Neurosurgery, Hokuto Social Medical Corporation Hokuto Hospital, 7-5 Inada, Obihiro, Hokkaido 080-0033 Japan; Department of Neurology, Hokuto Social Medical Corporation Hokuto Hospital, Obihiro, Hokkaido Japan; Department of Neurosurgery, Osaka City University Graduate School of Medicine, Osaka, Japan; Department of Neurosurgery, Ishikiriseiki Hospital, Higashiosaka, Osaka Japan

**Keywords:** Cavernous sinus dural arteriovenous fistula, Inferior petrosal sinus, Intravascular ultrasonography, Transvenous embolization

## Abstract

**Introduction:**

Although cavernous sinus (CS) dural arteriovenous fistulas (d-AVFs) are usually treated with transvenous embolization (TVE) via the inferior petrosal sinus (IPS), IPSs are sometimes thrombosed and angiographically invisible. In such cases, the first obstacle to TVE is detecting the entry to the IPS. We report a new technique for TVE via IPS using intravascular ultrasonography (IVUS).

**Methods:**

Three consecutive cases of CS d-AVF with ipsilateral or bilateral IPS occlusion were involved in this study. On TVE, the orifice of the IPS was investigated with IVUS placed in the jugular vein or jugular bulb.

**Results:**

This technique has been successfully adapted in all three cases. In two of these cases, IPS was well visualized with the help of IVUS, and TVE was successfully performed.

**Conclusion:**

To our knowledge, this is the first report to mention the usefulness of IVUS for detecting angiographically occult IPS.

## Introduction

Cavernous sinus (CS) dural arteriovenous fistula (d-AVF) is a condition in which the dural arteriovenous shunts form around the CS. This condition sometimes causes severe ocular symptoms and cerebral venous congestion. Although CS d-AVFs are usually treated with transvenous embolization (TVE) via the inferior petrosal sinus (IPS), IPSs are sometimes thrombosed and angiographically invisible. In such cases, the first obstacle to TVE is detecting the entry to the IPS, and this can prove difficult due to angiographic invisibility and anatomical variations. We describe herein the usefulness of intravascular ultrasonography (IVUS) for detecting an angiographically invisible caudal end of the IPS.

## Materials and methods

**Fig. 1:**
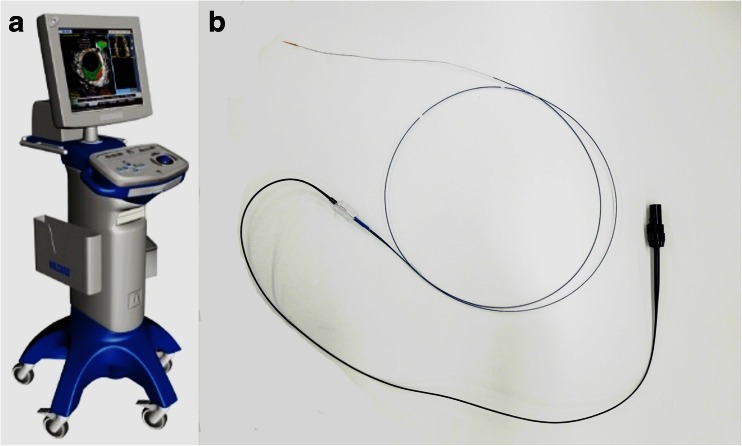
**a** A picture of the IVUS imaging system (Volcano S5™) and **b** the IVUS catheter (Eagle Eye Platinum®) used in our cases. The catheter is monorail type compatible with a 0.014-in. guidewire

Three consecutive cases of CS d-AVF with ipsilateral or bilateral IPS occlusion were involved in this study. In all cases, primary treatment was attempted by TVE via the occluded IPS, and attempts were made to detect the orifice of the IPS with IVUS (Eagle Eye Platinum®; Volcano, San Diego, CA) (Fig. 1).

## TVE procedure protocol

TVE procedures were performed under general or local anesthesia with femoral puncture. A 6-Fr guiding catheter was placed in the ipsilateral external or common carotid artery via a left transfemoral approach for control arteriography. Another 6-Fr guiding sheath was placed in the ipsilateral jugular vein (JV) via a right transfemoral approach. Initially, a microcatheter and 300-cm 0.014-in. microguidewire were navigated to the ipsilateral transverse sinus, then the microcatheter was withdrawn with an exchange technique, and the IVUS catheter was advanced along with the microguidewire as distal as possible. The IVUS catheter was slowly pulled, and a gray-scale image and ChromaFlo® image were obtained. The rostral half of the JV and, if possible, the jugular bulb (JB) were investigated using this technique. Under this guidance, we tried to navigate the microcatheter to the IPS and performed TVE in the standard manner.

In this study, whether the IVUS could pass through the JB and whether the IVUS could detect the caudal end of the IPS were evaluated retrospectively.

## Results

Among the three cases investigated, the tip of the IVUS catheter could not pass through the JB due to its tortuosity in case 1. In cases 2 and 3, the tip of the IVUS catheter was able to pass through the JB without difficulty. Although the JB and its surrounding structures were difficult to identify with IVUS in these two cases, the JV and surrounding tissue were clearly visualized with IVUS in all three cases.

In two of the three cases (cases 1 and 3), the caudal ends of the IPSs were clearly visualized with IVUS and ChromaFlo®. These two cases both had low-set IPSs originating from the extracranial JV. In both cases, with the aid of IVUS, a microcatheter was easily navigated to the orifice of the IPS and finally to the CS. In the remaining case (case 2), IVUS and ChromaFlo® failed to visualize the orifice of the IPS (Table 1). No procedure-related complications were encountered in any of the three cases.

**Table 1:** Summary of the three cases

	Visualization of JB and surrounding structures	Visualization of JV and surrounding structures	Visualization of IPS with gray scale	Visualization of IPS with ChromaFlo®	Pushability through JB	Origin of IPS
Case 1	NA	Comprehensible	Enable	Enable	Unable	Extracranial
Case 2	Incomprehensible	Comprehensible	Unable	Unable	Enable	Undetected
Case 3	Incomprehensible	Comprehensible	Enable	Enable	Enable	Extracranial

*NA* not available

## Case presentation

### Case 1

**Fig. 2:**
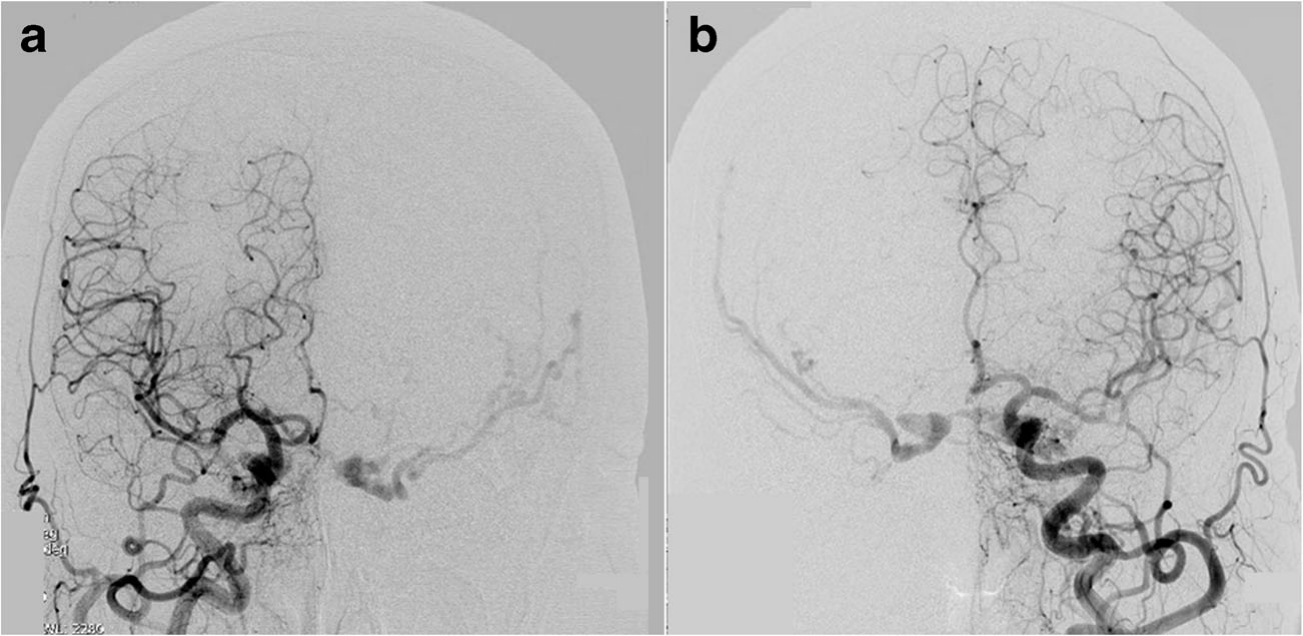
Diagnostic angiography in case 1. Bilateral (**a** right, **b** left) common carotid angiography demonstrates CS d-AVF involving bilateral CSs. Shunt blood flow mainly drains to bilateral SMCVs in a retrograde pattern. Bilateral IPSs are not angiographically visualized

The patient was a 73-year-old woman who developed pulsatile tinnitus and diplopia 3 months before admission. Two months later, although tinnitus had disappeared, she had developed pulsatile headache. Diagnostic angiography revealed bilateral CS d-AVF, supplied by the dural branches of the bilateral external and internal carotid arteries. Shunt blood flow mainly drained through bilateral superficial middle cerebral veins (SMCVs) in a retrograde pattern. Bilateral IPSs and superior orbital veins (SOVs) were not able to be visualized angiographically (Fig. 2).

**Fig. 3:**
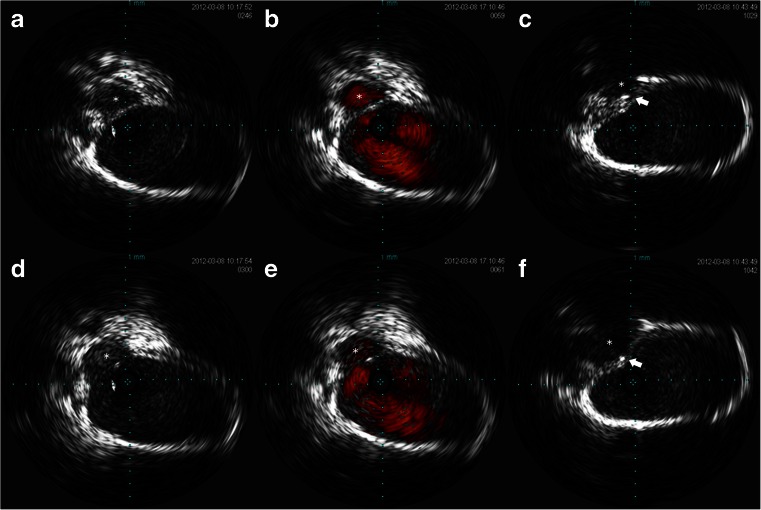
IVUS images in case 1. *Left* (**a**, **d**) gray-scale image. *Middle* (**b**, **e**) ChromaFlo® image. *Right* (**c**, **f**) gray-scale image after wire insertion. *Asterisks* (*) indicate IPS, and *white arrows* indicate the guidewire inserted into the IPS. The *upper three images* (**a**, **b**, **c**) represent slices slightly distal to the orifice of the IPS, with the IPS is running parallel to the JV. The *lower three images* (**d**, **e**, **f**) show slices at the orifice of the IPS

TVE through the left IPS was performed under general anesthesia in accordance with the protocol described above. In this case, the tip of the IVUS catheter could not pass through the JB due to its tortuosity. Almost the entire rostral half of the JV was investigated with IVUS, and the angiographically invisible IPS was visualized, identifying a low-set caudal end of the IPS. ChromaFlo® image could also detect the angiographically undetectable blood flow in the IPS (Fig. 3). Under this guidance, we were able to easily navigate the microcatheter to the IPS and finally to the CS. Initially, we aimed to occlude the shunt point selectively, but this proved to be difficult. We therefore performed sinus packing, and the shunt was then completely obliterated.

### Case 2

**Fig. 4:**
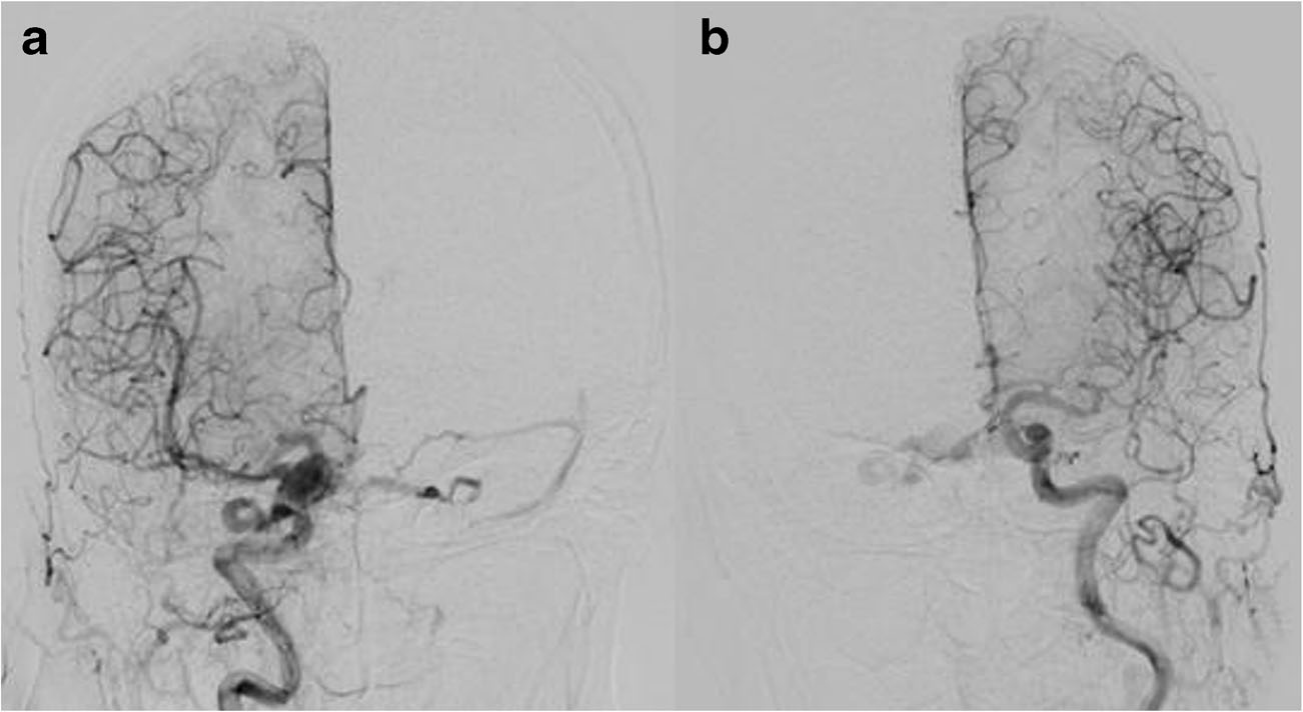
Diagnostic angiography in case 2. Bilateral (**a** right, **b** left) common carotid angiography demonstrates CS d-AVF involving bilateral CSs. Shunt blood flow mainly drains through bilateral SOVs in a retrograde pattern and partially draining into the leptomeningeal venous drainage of the posterior fossa. Bilateral IPSs are not seen angiographically

The patient was an 82-year-old woman. She developed diplopia and chemosis of both eyes 1 month before admission. Diagnostic angiography revealed CS d-AVF involving the CS bilaterally, supplied by the dural branches of the bilateral external and partially internal carotid arteries. Shunt blood flow mainly drained into bilateral SOVs in a retrograde pattern, with partial drainage through the retrograde leptomeningeal venous drainage to the posterior fossa. Bilateral IPSs were not able to be visualized angiographically (Fig. 4).

TVE through the right IPS was tried under local anesthesia in accordance with the protocol described. In this case, the tip of the IVUS catheter could pass through the JB without difficulty. Although the sigmoid sinus and JB were visualized with IVUS, the results were difficult to comprehend due to the surrounding bone structure and its tortuosity. On the other hand, the JV and surrounding tissue were clearly visualized with gray-scale and ChromaFlo® images, and the image was easily comprehended. In this case, we could not detect the entry of the IPS with IVUS. We subsequently searched for the orifice of the IPS in the usual manner for 1 h, but failed and abandoned TVE. Palliative transarterial embolization (TAE) was performed using poly vinyl alcohol (PVA) particles. After the procedure, symptoms improved.

### Case 3

**Fig. 5:**
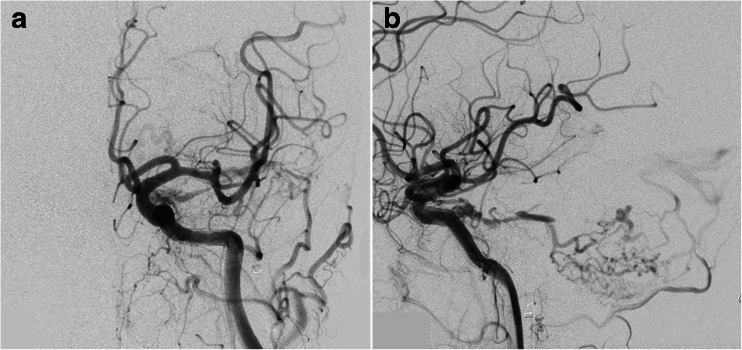
Diagnostic left common carotid angiography in case 3 (**a** AP view, **b** lateral view). CS d-AVF involves the left cavernous sinus and is supplied by the dural branches of the left external and internal carotid arteries. Shunt blood flow drains into the left petrosal vein via the left superior petrosal sinus in a retrograde pattern. The ipsilateral IPS is not angiographically visualized

The patient was a 73-year-old woman who had developed gait disturbance 2 weeks before admission. Diagnostic angiography revealed CS d-AVF involving the left cavernous sinus and supplied by the dural branches of the left external and internal carotid arteries. Shunt blood flow drained into the left petrosal vein via the left superior petrosal sinus in a retrograde manner. Ipsilateral IPS was not angiographically visualized (Fig. 5).

**Fig. 6:**
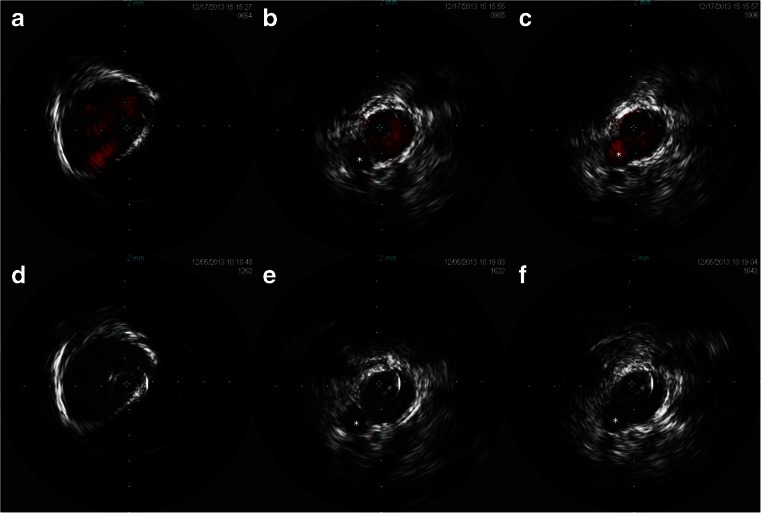
IVUS image in case 3. *Upper images* (**a**, **b**, **c**) gray-scale images. *Lower images* (**d**, **e**, **f**) ChromaFlo® images. *Right* (**a**, **d**) a slice of the JB, in which the vessel wall of the JB is visualized, but the surrounding tissue is not identifiable. *Middle* (**b**, **e**) a slice slightly distal to the orifice of the IPS, in which the IPS running parallel to the JV is clearly demonstrated in both images. *Left* (**c**, **f**) just the slice of the orifice of the IPS. *Asterisks* (*) indicate IPS

TVE through the left IPS was performed under general anesthesia using the described protocol. In this case, the tip of the IVUS catheter was also able to easily pass through the JB. Although the sigmoid sinus and JB were visualized on IVUS, comprehension of the results was difficult, as in case 2. The JV and surrounding tissue were again clearly visualized with gray-scale and ChromaFlo® images. In this case, the low-set entry of the IPS was clearly identified in gray scale and the angiographically undetectable blood flow in the IPS could be detected on ChromaFlo® images (Fig. 6). Under this guidance, we were able to easily navigate the microcatheter to the IPS and finally to the CS. Shunt blood flow was successfully obliterated.

## Discussion

To perform TVE via a thrombosed IPS, initially, the orifice of the angiographically invisible IPS must be located. Operators generally rely only on anatomical knowledge and palpation by hand to identify the IPS, but this is sometimes difficult due to its invisibility and anatomical variations.

Several reports have described anatomical variations to the caudal end of the IPS [1, 2]. Mitsuhashi et al. [2] recently investigated this variation with rotation digital-subtraction venography and proposed a new classification. According to their study, 3.6 % of cases showed no direct connection between the IPS and the JV or JB (type E), and 16.9 % showed no IPS (type F) [2]. With these types, detecting the entry point to the occluded IPS with standard techniques might be impossible or very difficult. If we could determine whether a direct connection between JV and IPS existed, we would be able to save time and avoid unnecessary irradiation. We therefore considered new methods for detecting the invisible origin of the IPS.

At the end of the 1980s, IVUS was introduced as a new modality to visualize vessel walls and their characteristics [3, 4]. Since then, this modality has predominantly been used for atherosclerotic stenosis or arterial dissection in interventional radiology and cardiology [5–10].

Some reports have described other usages of IVUS. Nishio et al. reported the utility of IVUS for monitoring herniated coils in the embolization of direct carotid cavernous fistula [11]. Yoshioka et al. also reported TAE of direct vertebral arteriovenous fistula with the aid of IVUS [12]. In the field of coronary intervention, some technical reports have described chronic total occlusion in which target vessels were visualized with the IVUS catheter placed in other vessels [13, 14].

IVUS can visualize not only the vessel wall but also the surrounding structures. This property of IVUS has been applied to the techniques described by other investigators. Our technique also followed this concept.

In this study, IVUS was able to visualize the JV and surrounding tissue clearly in all three cases and could detect the extracranial origin of the IPS in two cases. In those two cases, IVUS could also detect the angiographically invisible blood flow in the caudal end of the IPS with ChromaFlo®. IVUS might represent a useful modality for detecting an extracranial origin of the IPS.

On the other hand, the JB may sometimes be difficult to pass through with IVUS catheter for its tortuosity. In one of our three cases, we could not navigate the tip of IVUS catheter to the sigmoid sinus. In such a case, a stiffer guiding catheter or direct puncture of JV might be needed to pass through the tortuous JB. In the remaining two cases, navigation of the tip of the IVUS catheter to the sigmoid sinus was easily carried out even under local anesthesia. Although the JB was investigated with IVUS in those two cases, IVUS images were difficult to interpret in this portion. The JB is anatomically tortuous and covered with bone. When we pulled back the IVUS catheter, as the tip was caught by the tortuosity of the JB, moving the tip in a smooth manner proved difficult. The surrounding bone interfered with clear vision with its acoustic shadow. Moreover, as the JB has multiple branches, distinguishing the IPS from other branches such as the marginal sinus, posterior condylar vein, and petroclival vein might be difficult with IVUS. From these findings, IVUS might not be particularly useful for detecting an intracranial origin of the IPS. At present, this might represent a major limitation of our technique. To overcome this limitation, other methods such as optical coherence tomography or high-frequency IVUS might be applicable.

Our technique thus remains incomplete in terms of reliably detecting IPS in all cases. However, according to the report by Mitsuhashi et al., the IPS originates from the extracranial JV in about 70 % of cases, so if an extracranial origin of the IPS could be reliably detected with this technique, at least 70 % of cases might benefit [2]. IVUS would be one useful option for such cases.

## Conclusion

This is the first report to describe the use of IVUS for detecting an occluded IPS. Although IVUS could detect a low-set IPS, detecting an intracranial origin of the IPS is difficult with this technique. As this report involves only a small number of cases, and represents only a preliminary experience, accumulation of more cases will clarify whether this technique is useful.
